# A Miniaturized Extruder to Prototype Amorphous Solid Dispersions: Selection of Plasticizers for Hot Melt Extrusion

**DOI:** 10.3390/pharmaceutics10020058

**Published:** 2018-05-19

**Authors:** Matthias E. Lauer, Reto Maurer, Anne T. De Paepe, Cordula Stillhart, Laurence Jacob, Rajesh James, Yuki Kojima, Rene Rietmann, Tom Kissling, Joost A. van den Ende, Sabine Schwarz, Olaf Grassmann, Susanne Page

**Affiliations:** 1 Roche Pharmaceutical Research and Early Development (pRED), Roche Innovation Center Basel, F. Hoffmann-La Roche Ltd., CH-4070 Basel, Switzerland; anne.de_paepe@roche.com (A.T.D.P.); rajesh.james.ch@gmail.com (R.J.); rietmann.rene@gmail.com (R.R.); tom.kissling@roche.com (T.K.); joost_adam.van_den_ende@roche.com (J.A.v.d.E.); Sabine.Schwarz.ss1@roche.com (S.S.); 2 Pharmaceutical Research and Development, F. Hoffmann-La Roche Ltd., CH-4070 Basel, Switzerland; Reto.Maurer@roche.com (R.M.); cordula.stillhart@roche.com (C.S.); Laurence.Jacob@roche.com (L.J.); 3 Production Engineering Department (Formulation Technology), Chugai Pharmaceutical Co., Ltd., 5-1 Ukima 5-chome, Kita-ku, Tokyo 115-8543, Japan; kojimayuk@chugai-pharm.co.jp; 4 Pharma Technical Development, F. Hoffmann-La Roche Ltd., Grenzacherstrasse 124, CH-4070 Basel, Switzerland; Olaf.Grassmann@roche.com

**Keywords:** hot melt extrusion, nanotechnology, torque rheometer, amorphous solid dispersion

## Abstract

Hot-melt extrusion is an option to fabricate amorphous solid dispersions and to enhance oral bioavailability of poorly soluble compounds. The selection of suitable polymer carriers and processing aids determines the dissolution, homogeneity and stability performance of this solid dosage form. A miniaturized extrusion device (MinEx) was developed and Hypromellose acetate succinate type L (HPMCAS-L) based extrudates containing the model drugs neurokinin-1 (NK1) and cholesterylester transfer protein (CETP) were manufactured, plasticizers were added and their impact on dissolution and solid-state properties were assessed. Similar mixtures were manufactured with a lab-scale extruder, for face to face comparison. The properties of MinEx extrudates widely translated to those manufactured with a lab-scale extruder. Plasticizers, Polyethyleneglycol 4000 (PEG4000) and Poloxamer 188, were homogenously distributed but decreased the storage stability of the extrudates. Stearic acid was found condensed in ultrathin nanoplatelets which did not impact the storage stability of the system. Depending on their distribution and physicochemical properties, plasticizers can modulate storage stability and dissolution performance of extrudates. MinEx is a valuable prototyping-screening method and enables rational selection of plasticizers in a time and material sparing manner. In eight out of eight cases the properties of the extrudates translated to products manufactured in lab-scale extrusion trials.

## 1. Introduction

Amorphous solid dispersions (ASDs) are an option to increase the oral bioavailability of poorly soluble drug crystals [1]. The rationale is to disperse and trap dissolved-single molecules in a polymer carrier matrix and to bypass the energy required to dissolve entire crystals [2]. ASDs are complex and metastable materials and the drug molecules can crystallize upon storage. To date, ASDs are not a standard formulation principle, in both, preclinical and clinical stages of development. ASDs also have advantages over crystalline dosage forms. Unfavourable mechanical aspects of crystalline compounds can be tuned or optimized by selecting suitable polymers and ingredients and more importantly, a kinetically stable ASD is unlikely to turn into an unknown polymorphic form [3].

Various assays have been established to screen for polymer carrier systems which combine best drug dissolution enhancement with excellent solid-state properties, such as high drug polymer miscibility and storage stability [4,5]. Quench-cooled melt mixtures (QCMMs) are an established preparative-explorative platform to assay molecular miscibility and physical stability of ASDs [6]. QCMMs can be explored with scanning probe microscopy (SPM) for structure-property relations on molecular length scales. The determination of molecular surface roughness with atomic force microscopy (AFM), AFM is the most established variant of SPMs, is an objective way to judge the molecular dispersity of ASDs. The high spatial resolution of the method enables studying of de-mixing and crystallization processes on molecular time scales. Therefore, stability studies can be carried out within hours, instead of within weeks [7,8]. Beyond mapping of spatial drug-polymer distributions, SPMs enable to concertedly probe spectroscopic, mechanical and thermal properties and thereby enlighten complex structure-property relations in a direct manner [9,10]. A disadvantage of SPMs is that only smooth and small surface areas are accessible with the SPM sensor.

The final performance of ASDs does not only depend on the intrinsic attributes of the components but also on the technical processes used to create the dispersion. Besides spray drying (SD) the process of hot melt extrusion (HME) is a key technology to enable ASDs [4,11]. Whereas SD gives micrometre-sized particles with low bulk density, the HME process produces dense ‘spaghetti’-shaped strands on the millimetre scale [12]. A systemic disadvantage of HME is the high thermal and/or mechanochemical stress which is often necessary to blend viscous components [13,14,15]. Processing aids, plasticizers, reduce the viscosity of ASDs, however, as they typically remain in the formulation they may also modulate stability and dissolution performance of the final product. 

Plasticizers modulate the physical interaction, persistence and conformational freedom of macromolecules and their supramolecular networks, the presence of flexible-fluidic and plasticizing molecules soften intra- and intermolecular polymer chain interactions. Moreover, other modes of plasticizing actions have been described, for example lubricity theory, gel theory, free volume and mechanistic theory [16,17]. Torque rheometry is an established technique to measure and screen for suitable plasticizers and conditions, also, the method enables to predict a suitable processing-temperature range [18,19]. Plasticizers are often low molecular weight compounds, such as citrate esters, fatty acid esters, sebacate esters, phthalate esters, glycerol derivatives, or vitamin E and more [12]. The incorporation of pressurized CO_2_, for example, enlarges the free volume and lowers the amount of intermolecular contacts. The result is a lower glass transition temperature which is beneficial for the extrusion process. The resulting foam-like extrudates can then easily be downstream processed to a tablet [20,21]. The development and manufacturing of ASDs ravels into a multivariate challenge, it often requires time and material consuming trials until proper ingredients and processing parameters are set.

Here, we explore a novel and practical prototyping device, the Miniaturized Extruder (MinEx) to achieve this goal. It is used in combination with torque rheometry to explore the impact of various plasticizers on processability as well as on dissolution and solid-state properties of ASDs in an efficient and elegant manner. As first model compounds, we selected neurokinin-1 receptor antagonist (NK1) and cholesterylester transfer protein inhibitor (CETP). Hypromellose acetate succinate type L (HPMCAS) was selected as polymeric carrier system. Polyethyleneglycol 4000 (PEG4000), Poloxamer 188 (Pol188) and stearic acid (SA) were used as plasticizer.

## 2. Materials and Methods

### 2.1. Materials

For this study two different active pharmaceutical ingredients (API), namely NK1 receptor antagonist (NK1, lot BS0009S004 and BS0009S005) and CETP Inhibitor (CETP, lot BS0605S002), were selected as model compounds. Both APIs were manufactured by F. Hoffmann-La Roche Ltd. (Basel, Switzerland). As polymer hydroxypropyl methylcellulose acetate succinate, type L from Shin-Etsu (Tokyo, Japan, HPMCAS, type LF: lots 9083207 and 2063182, HPMCAS, type LG: lot 0073198) was used as it represents a group of polymers which is difficult to process via hot melt extrusion due to high melt viscosity. Depending on the experiments performed, either HPMCAS LF or a mixture of 30% HPMCAS LF (micronized grade) and 70% HPMCAS LG (granular grade) was used in order to improve the flowability into the extruder. The plasticizers used in this study were taken from different chemical classes and were used as plasticizer before [22,23,24,25,26,27,28]. In total three different plasticizers, polyethylenglycol (PEG4000) from Fluka (Buchs, Switzerland, lot 121490822805P02), Poloxamer 188 from BASF (Ludwigshafen, Germany, lot WO45740) and stearic acid from Lipid Chem Sdn Bhd (Masai, Malaysia, lot LSB/10-2796), were used.

### 2.2. Miniaturized Extrusion

**Figure 1:**
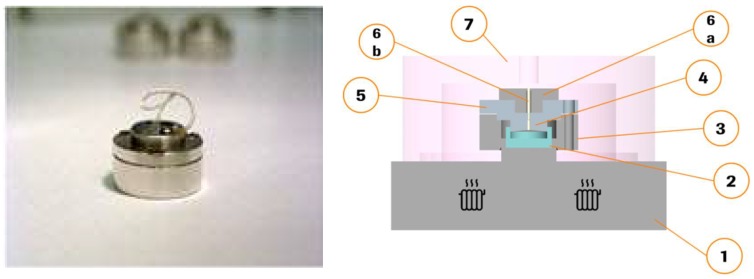
Device used for miniaturized extrusion (**left** photography, **right** schematic representation). 1—Hot stage, 2—Container, 3—Chassis, 4—Piston, 5—Cover, 6a—Nozzle, 6b—Tubing, 7—Vacuum cap.

Miniaturized extrusion was carried out in a newly developed device (Figure 1) which has been designed by the F. Hoffmann-La Roche machine shop (Basel, Switzerland), the construction plans are shown within the Appendix. Part 1, 2, 4, 6a and 7 are made from aluminium, part 3 from titan and 6b from poly tetra fluoro ethylene (PTFE). It enables preparation of small quantities, in the range of typically 30–35 mg extrudates.

Preparation of solvent cast films: In order to ensure a good homogeneity of the mixture prior to extrusion the following sample preparation was used. In this study, the same compositions (Table 1) were used for all extrusion trials (miniaturized and laboratory scale). All single components were accurately weighted (total amount 100 mg) and dissolved in organic solvent (Acetone-Ethanol-mixture 1:1) at a concentration of 50 mg/mL. The solutions were homogenized by stirring at room temperature until all components were fully dissolved. 1 mL of the obtained solution was placed drop-wise on a PTFE (Polytetra GMBH, Moenchengladbach, Germany) foil, which was rinsed with isopropanol for cleaning purposes and annealed to *T* = 70 °C, in order to prepare a solvent cast film. After few minutes of drying (IKA RCT basic, Staufen, Germany) at 70 °C, a flexible-jelly material was obtained, complete drying of these films was avoided. The films reached a rubber-like state and could be easily peeled off from the PTFE foil and transferred into the container of the MinEx extrusion device. After the films had been successfully placed in the containers they were stored for at least 12 h in a drying chamber, at *T* = 40 °C for drying purposes.

**Figure 2:**
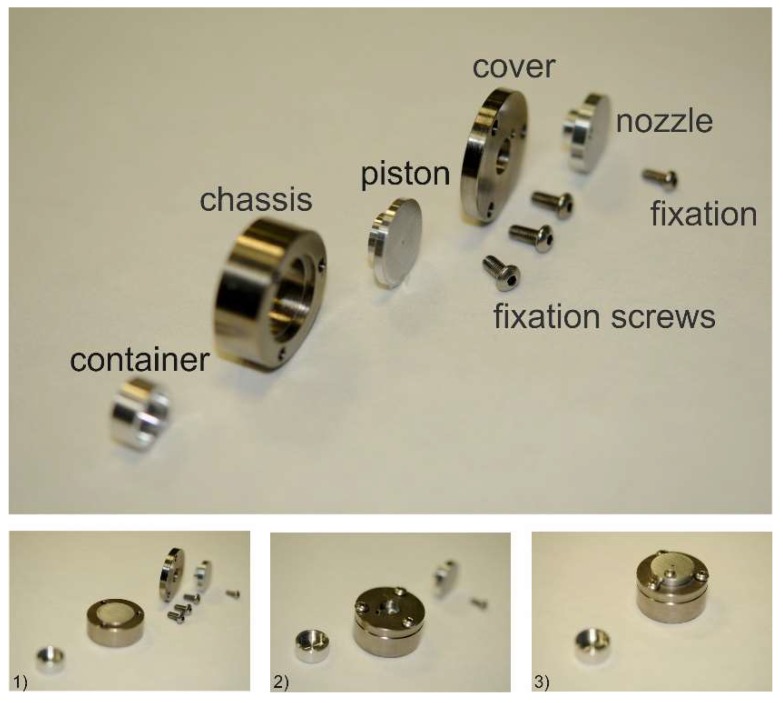
Photographs show building blocks and assembly of the extrusion nozzle. The piston is mounted in the chassis (**1**), and covered with three fixation screws (**2**), finally the nozzle needs to be fixated (**3**) Assembly of the MinEx extrusion nozzle.

**Figure A1:**
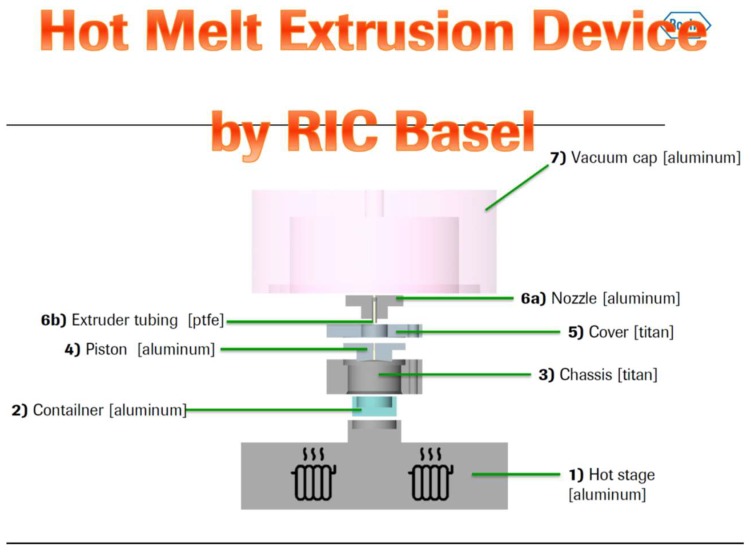
Building blocks of the Mini-Extruder.

**Figure A2:**
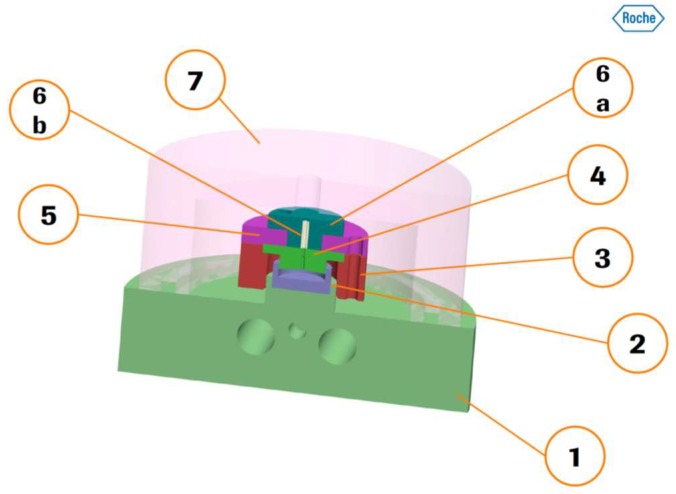
3d construction drawing of parts already shown in Figure A1. The part numbers match with construction drawing given in Figure A3.

**Figure A3:**
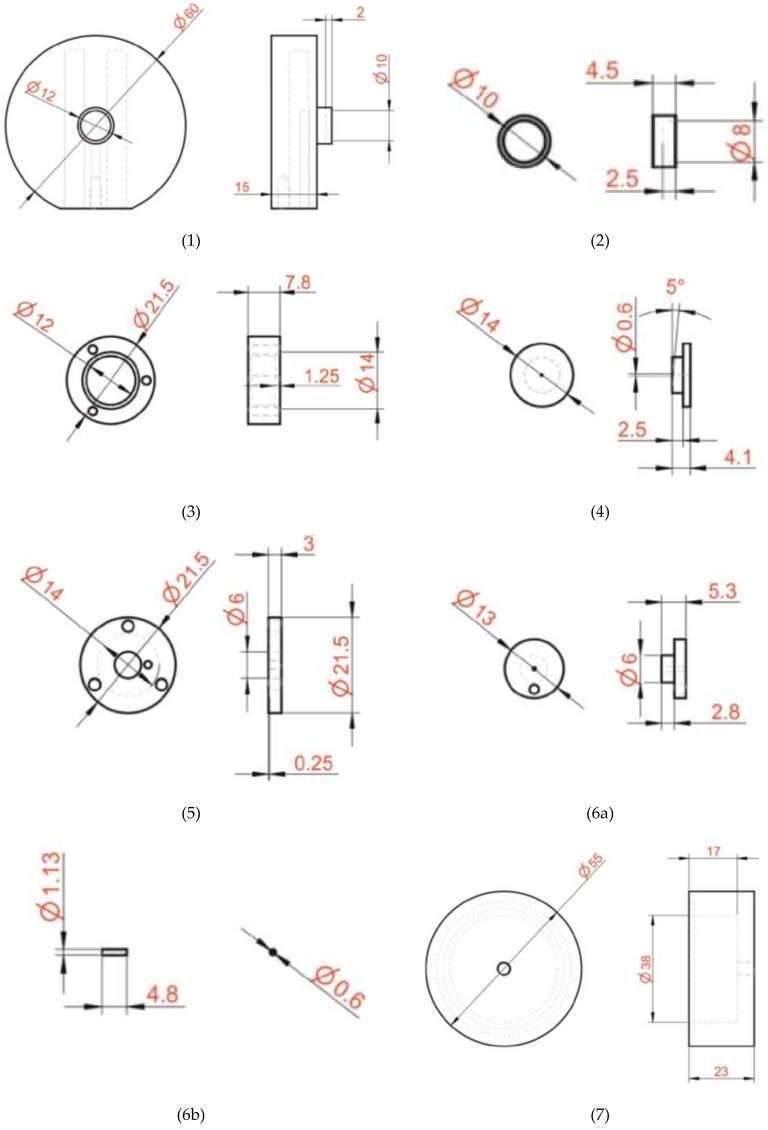
True to scale representations, in millimetres: hot stage plate (**1**), container (**2**), cover (**3**), piston (**4**), chassis (**5**), Nozzle (**6a**), Teflon tubing (**6b**), vacuum cap-(**7**).

Assembly and extrusion with the MinEx device: The construction plans of all MinEx building blocks are shown in the Appendix, (Figure A1, Figure A2 and Figure A3). Each extrusion nozzle was assembled from four separate main parts. The ring-base was first locked with the piston. An adaptor ring was then fixed with three screws on the base; the extrusion nozzle was mounted on top of all and fixed by use of a single and slightly smaller screw. The filled and well-dried containers were placed on the heating stage and closed with the extrusion nozzle. The extrusion nozzle plunged the container (Figure 2). This whole arrangement was then covered and sealed with a vacuum cap, brought to high vacuum and slowly heated. At a temperature of *T* = 140 °C the cap was flooded with air and removed. At *T* = 160 °C the extrusion was initiated by a hand press; the extrusion nozzle was pressed against the container to confine and to extrude a viscous melt. The material was cooled at room temperature. The obtained miniaturized extrudates were characterized by atomic force microscopy (AFM), X-ray powder diffraction (XRPD), differential scanning calorimetry (DSC), fourier transformed infrared spectroscopy (FTIR) and dissolution testing.

### 2.3. Investigating the Melt Behaviour

The melt behaviour of the binary or ternary mixtures (see Table 1) was investigated using the Lab-Station Plasti-Corder^®^ Torque Rheometer (Brabender^®^ GmbH & Co., KG, Duisburg, Germany). Initially, the temperature and screw speed were set to 30 °C and 2 rpm, respectively. The powder blend (40 g) was added gradually into the torque rheometer within 10 min. The equipment was closed with a 5 kg weight and the screw speed was increased to 20 rpm immediately, while the temperature was increased to 140 °C at a heating rate of 10 K/min. Afterwards, temperature and screw speed were kept constant for 10 min. The specific energy at steady state (in J/g) was calculated according to Equation (1):

(1) $$SE_{\mathrm{SS}}=\frac{Pm\Delta t}{m}$$

(2) $$P_{m}=2\pi nM_{\mathrm{ss}}$$

where *M*_ss_ is the torque on the screw shafts at steady state (average torque from the last 10 min (=Δt) of the experiment in Nm), *n* is the screw speed in s^−1^, *P*_m_ is the mechanical power in Nm/s, Δt is the measuring time interval in s and m is the sample weight in g.

### 2.4. Manufacturing of Extrudates

Extrudates were manufactured using a lab-scale extruder TSE 12 X 36D (Brabender^®^ GmbH & Co., KG, Duisburg, Germany) equipped with two similar screws containing conveying as well as kneading elements (two forward kneading zones and a 90° kneading zone providing melt seal). The screw speed was set to 50 rpm and the powder blend was fed into the extruder at a rate of 1.0 to 1.2 g/min. The temperature in the feeding zone was set to 80 °C, whereas in all other zones it was set to 165 °C. The composition of the extrudates is given in Table 1. The specific mechanical energy input (SME) was calculated according to Equation (3):

(3) $$SME=\frac{MNP}{N_{r}m}\;$$

where *M* is the motor load at steady state, *N* is the actual screw speed in rpm, *P* is the rated power in kW (6.13 kW for the extruder used), *N*_r_ is the rated screw speed in rpm (600 rpm for the extruder used) and m is the mass flow in kg/s. In addition to the specific mechanical energy also the temperature in the die (T_die) and pressure at die (p___die) were compared. The extrudates were milled using an IKA mill (IKA^®^-Werke GmbH & Co., KG, Staufen, Germany) and screened manually through a 1.0 mm screen. For drug release testing the sieve fraction 250 to 500 μm was used.

### 2.5. Analytical Methods for Characterization

#### 2.5.1. Atomic Force Microscopy (AFM)

**Preparation of extrudates for AFM investigations:** For microscopic investigations fresh and smoothly fractured extrudate surfaces are required. Planar fracture surfaces are mandatory for meaningful AFM measurements and were obtained by introducing predetermined fracture points on the outer surfaces of the spaghetti-shaped extrudates. A razor blade was carefully pushed onto the surface of the spaghetti-shaped extrudates, the extrudates were then carefully scrolled to generate a defined scratch with a depth of ~0.5 mm. Fracture was introduced by bending the extrudates with the use of two tweezers. The freshly fractured extrudates were mounted on an optical glass slide by use of a 2-component epoxy resin, which hardened within ~5 min. Before the hardening reaction had been completed the extrudate orientation was corrected to get the fracture surface as horizontal as possible. This step is mandatory to enable non-destructive imaging and automated sample changing within Atomic Force Microscope operations. 

**Atomic Force Microscopy:** Freshly fractured extrudates on microscopic glass slides were mounted on the micrometre positioning stage of a Dimension Icon AFM (Bruker, Santa Barbara, CA, USA). Between 10 and 25 regions per sample were programmed to be automatically characterized using the software routine “programmed move” in Tapping Mode (Bruker Nansocope V8 r1.5). Height, phase and amplitude images were collected simultaneously, using etched silicon cantilevers with a nominal spring constant of *k* = 20–80 N/m (Bruker AFM Probes, RTESPA) for MIMix homogeneity analyses and of *k* = 4 N/m (Bruker AFM Probes, RFESPA) for recording phase contrast maps on samples after stress storage [7,8]. The typical free vibration amplitude was in the range of *A* = 10 nm (RTESPA) and *A* = 30 nm (RFESPA), the images were recorded with set-point amplitudes corresponding to 60–70% of the free amplitude. Image areas of 3 × 3 µm^2^ were recorded at a resolution of 768 × 768 pixels. All data were batch-processed using Scanning Probe Image Processor, SPIP 5.1.1 (Image Metrology, Horsholm, Denmark,). Height data were plane-corrected by applying a 3rd order polynomial fit. The phase images shown are non-processed raw data.

Stability studies were carried out to assess and compare the stability of the produced lab-scale and MinEx extrudates. Freshly fractured extrudates were stored at accelerated stability conditions (*T* = 40 °C, RH = 75%) for 2 h. Before stress storage the mounted extrudates were brought to a temperature of *T* = 40 °C to minimize the risk of unspecific moisture condensation during incubation. The surfaces were stressed in a pre-equilibrated desiccator and finally quickly removed from the humid, high temperature environment and “dried” in a vacuum chamber.

#### 2.5.2. Differential Scanning Calorimetry

Thermoanalytical characterizations of polymers, APIs and plasticizers and the corresponding extrudates were performed using a differential scanning calorimeter from Mettler Toledo, Greifensee, Switzerland. Each measurement was performed with approximately 4 mg of the material which was placed into a 40 µL crucible. In order to determine the melting point (Tm) or the glass transition temperature of excipients and APIs, the substances were heated from 25 °C to a temperature above the melting point of the APIs (140 °C for NK1 and 155 °C for CETP), to at least 220 °C in case of the plasticizers and the polymer at a rate of 10 K/min in order to determine the onset of the melting point (*T*m) or the midpoint of the glass transition temperature (*T*g). These investigations were performed in duplicate. 

The initial physical state of samples manufactured by MinEx and lab-scale extrusion was determined by heating the sample from 20 to 180 °C at a heating rate of 10 °C. The same method was applied in order to investigate material from stability assessments (single measurement). Pre-testing has shown that the APIs are able to partially dissolve in the molten matrix containing the polymer with or without plasticizer (data not shown) therefore DSC was not used in a quantitative manor.

In addition, the re-crystallization behaviour of the APIs and plasticizers was tested using a method described by Baird et al. before [29]. The sample was initially heated from 25 to 170 °C with a heating rate of 10 K/min (phase 1). It was kept constant for 170 °C for 3 min (phase 2), before it was cooled down to −75 °C at a rate of −20 K/min (phase 3). In the last part, the sample was reheated to 160 °C at a rate of 10 K/min (phase 4). Depending on the occurrence of a melting peak the substances are classified into fast (freezing peak in phase 3), medium (melting peak in phase 4) and slow crystallizer (no freezing/melting peak in phase 3 & 4).

#### 2.5.3. Fourier Transformed Infrared Spectroscopy (FTIR)

The attenuated total reflection (ATR) FTIR spectra were recorded using a ThermoNicolet iS5 FTIR spectrometer (Thermo Fisher Scientific, Madison, USA) with single bounce diamond ATR accessory. The spectral range is between 4000 and 650 cm^−1^, resolution 2 cm^−1^ and 50 scans were collected.

#### 2.5.4. X-ray Powder Diffraction (XRPD)

X-ray diffraction patterns were recorded at ambient conditions in transmission geometry with a STOE STADI P diffractometer (Fa STOE & Cie. GmbH , Darmstadt, Germany ) Cu Ka radiation, primary monochromator, silicon strip detector, angular range 3° to 42° 2Theta, approximately 30 min total measurement time). The samples were prepared and analysed without further processing (e.g., grinding or sieving) of the substance.

#### 2.5.5. Two-Stage Dissolution Testing 

The drug release kinetics out of extrudates was characterized using a small-scale dissolution test. The dissolution test included two stages to simulate the gastrointestinal transit of the formulations. The extrudates were first stored in simulated gastric fluid (SGF pH 2) for 30 min and afterwards in fasted-state simulated intestinal fluid for 2 h (FaSSIF pH 6.5). SGF consisted of a 60 mM sodium chloride solution at pH 2. FaSSIF pH 6.5 was composed from 106 mM NaCl, 28.6 mM NaH_2_PO_4_, 3 mM sodium taurocholate and 0.75 mM soybean lecithin [30]. The 250 to 500 µm sieve fractions of lab-scale extrudates were used for dissolution testing and the experiments were carried out in triplicate. Dissolution testing with MinEx samples was conducted with the unmilled extrudates, because the amount of material was too small for an additional milling step.

An aliquot of extrudate corresponding to 6 mg API was weighted in a glass vial. To initiate the dissolution test, 1.5 mL SGF pH 2 were added and the vial was incubated on a rotating shaker (GFL 3025, Gesellschaft für Labortechnik mbH, Burgwedel, Germany) at 5 rpm and 37 °C. These conditions were applied to simulate gastric motility and temperature. A 167 µL sample was withdrawn after 10, 20 and 30 min and immediately centrifuged to separate undissolved drug (13,000 rpm, 90 s). The supernatant was diluted in acetonitrile prior to high performance liquid chromatography (HPLC) analysis. After 30 min of dispersion in SGF, 3 mL of a FaSSIF pre-concentrate were added to the SGF phase. This pre-concentrate contained a higher concentration of bile salts and lecithin, in order to obtain FaSSIF upon dilution in SGF. The vials were again incubated at 5 rpm and 37 °C and a 200 µL sample was removed after 5, 10, 20, 35, 45, 60, 120, 240 min. Samples were centrifuged (13,000 rpm, 90 s) and the supernatant was diluted in acetonitrile prior to HPLC analysis. 

HPLC analysis was done using a Waters 2690 Alliance^®^ Separations Module, a 2487 Dual λ absorbance detector and a Waters XTerra^®^ MS C18 3.5 µm column (Waters Corp., Milford, MA, USA). The elution conditions for NK1 and CETP are detailed in Table 2.

### 2.6. Stability Testing

The milled extrudates were stored open at 40 °C/75%RH, 40 °C/25% RH and 25 °C/60%RH for 3, 6 and 12 months, respectively. Samples were taken and analysed using DSC and XRPD. Selected samples were analysed by FTIR ATR.

## 3. Results

### 3.1. Selection and Properties of Compounds

**Figure 3:**
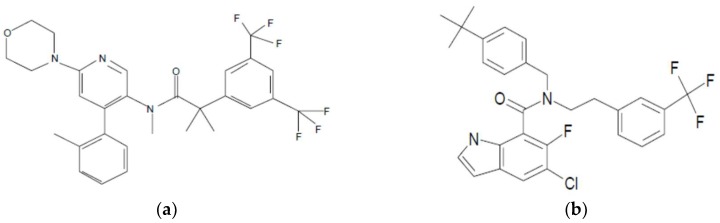
Chemical structure of neurokinin-1 receptor antagonist (NK1) (**a**) and cholesterylester transfer protein inhibitor (CETP) (**b**).

Two active pharmaceutical ingredients (APIs) were selected (Figure 3). NK1 (Figure 3a) has a molecular weight of *M* = 566 g/mol, a melting point of 130 °C and is classified as a slow re-crystallizer using the method described by Baird et al. [29]. NK1 can act as a hydrogen bond acceptor via a carbonyl group. The second API, CETP, has a molecular weight of *M* = 531 g/mol, a melting point of 146 °C and is a slow re-crystallizer. CETP has an amine and a carbonyl group and is a potential H-bond donor/acceptor.

HPMCAS was selected because it was found suitable for manufacturing and stabilization of HMEs in previous studies [7,8]. The polymer has a molecular weight of about 122 to 146 kDa [31] and its glass transition temperature (*T*g) amounts ~120 °C. The melt viscosity of the polymer in the temperature range of 160 to 180 °C is high as confirmed by the zero rate viscosity determined by Sarode et al. [32]. HPMCAS can still be processed within the technical limits of an extruder at temperatures below 190 °C but it is considered to be thermally labile at higher temperatures [33]. HPMCAS is a hydrogen acceptor and donor [34].

Three different plasticizing agents were selected. PEG4000 has a molecular weight between 3 and 4.8 kDa, the melting point onset is between *T* = 56 and 59 °C. Pol188 has a molecular weight between 7.7 and 9.5 kDa and a comparable melting point onset of 51 °C. As low molecular weight plasticizer we selected SA, it has a molecular weight of *M* = 284 Da, its melting point onset is *T* = 67 °C. All three plasticizers are known to be fast re-crystallizers [29]. SA condenses in lamellar platelets as they are typical for fatty acids [35].

### 3.2. Torque Rheometery to Determine Impact of Plasticizers on Viscocity and Melt Behaviour

**Figure 4:**
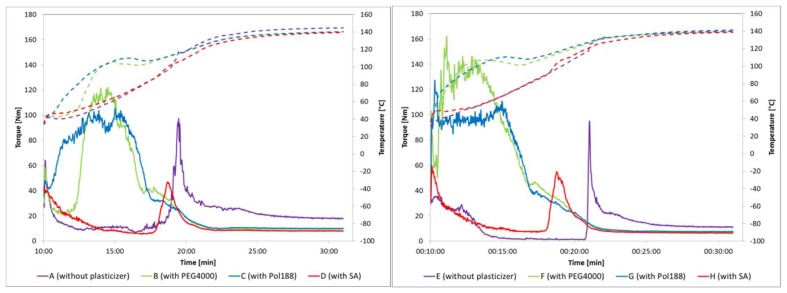
Results from torque rheometer measurements for NK1 (**left**) and CETP samples (**right**). Binary systems with Hypromellose acetate succinate type L (HPMCAS) (A,E) and ternary systems with HPMCAS + PEG4000 (B,F), with HPMCAS + Pol188 (C,G) and with HPMCAS + SA (D,H). The torque in NM is represented by the full line, whereas the temperature in °C is presented by the dotted line.

The melting viscosity behaviour of physical powder mixtures was explored using torque rheometry (Figure 4) because the MinEx device does not allow to assess such parameters. The results are summarized in Table 3. The powder components were fed into the measuring chamber within 10 min. 

In the absence of plasticizers, after a pre-conditioning phase, the torque levels off and becomes comparable and constant for both binary API mixtures. Upon heating to *T* = 120 °C, which is close to the *T*g of HPMCAS, the torque increases from ~10 to ~100 Nm and finally levels off to 20 Nm at steady state. The specific energy at steady state (*SE*_SS_) is summarized in Table 3. The *SE*_SS_ is lower in the presence of CETP than NK1, indicating that CETP has larger plasticizing effect than NK1.

Similar experiments were conducted with ternary mixtures and showed that all plasticizers beneficially modulate the viscosity of both API-HPMCAS systems. PEG4000 and Pol188 thereby lead to a comparable and significant torque-temperature shift, probably due to the low melting temperature of the compounds (59 °C and 51 °C). Heating to 110 °C significantly decreases the material viscosities and the final torque at steady state was about two times lower than in absence of plasticizers. 

SA also modulated the torque-temperature profile. The situation is qualitatively and quantitatively different than in presence of PEG4000 or Pol188. After the preconditioning phase the torque decreases, then, the profiles stay qualitatively comparable with mixtures lacking plasticizers. However, a significantly lower torque and moderate shift of the viscosity maximum to lower temperatures in comparison to the binary API-polymer systems is observed (47 to 55 Nm compared to 100 to 120 Nm). The microscopic mode of plasticizing action is indicated to be different than in cases with PEG4000 and Pol188 respectively.

### 3.3. Preparation of Lab-Scale and MinEx Extrudates

Eight compositions, four for each API, were manufactured containing API and polymer in a 3:7 w/w ratio and a plasticizer to polymer in a 1:9 *w/w* ratio. Two different extrusion techniques were applied; a lab-scale process and extrusion with the MinEx device.

**Lab-scale manufacturing of extrudates using the Brabender Lab-scale TSE 12/36:** The specific mechanical energy at steady state, which was calculated based on the motor load determined at steady state, the actual screw speed and the mass flow for both APIs, is summarized along with the pressure and temperature at die in Table 3. The specific mechanical energy of the API-polymer system is significantly lower for systems with CETP if compared with NK1. CETP has better plasticizing properties than NK1 in systems with HPMCAS, the result confirms observations made with torque rheometry before. Upon addition of plasticizers a further decrease in the specific mechanical energy is observed, which is in line with observations made with the torque rheometer (Figure 4 and Table 3). The selected plasticizers modulate the process in an anticipated manner.

**MinEx** is a novel extrusion platform to prototype extrudates of high dispersity, tens of milligrams can be produced at once. The device and preparative workflow is described in Material and Methods and only briefly summarized here: (1) casting media were prepared by dissolving and mixing of ingredients in acetone. (2) Flexible films on PTFE foils were prepared by solvent casting at temperature of *T* = 70 °C. The films were briefly dried to peel off foils without fracture. The films were transferred and pushed into the MinEx extrusion container. (3) The containers were closed with the MinEx extrusion nozzle. Residual solvent was removed by evacuating and drying of the assembled nozzle over night at *T* = 40 °C. (4) The assembled-loaded MinEx device was placed under a knuckle joint press and heated until the desired extrusion temperature of *T* = 160 °C had been reached. (5) At 160 °C, the knuckle joint press was both pushed downwards and a cylindrically shaped extrudate evolved and finally protruded the extrusion nozzle. (6) The device and extrudates were cooled on a massive steel plate and subjected to analytical characterization at ambient temperature. 

### 3.4. Nanostructure of MinEx and Lab-Scale Extrudates

**Figure 5:**
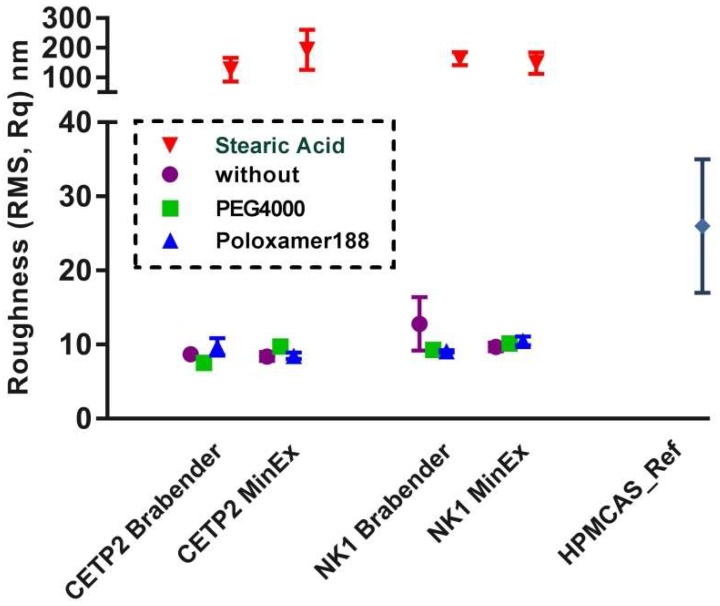
Molecular surface roughness of amorphous solid dispersions (ASDs) prepared with MinEx and with the lab-scale process, in presence and absence of plasticizers: ternary mixtures with Pol188 are shown with blue dots, with PEG4000 in green, with SA in red and all binary mixtures are shown in purple. A HPMCAS-reference extrudate which has been prepared with MinEx is shown at the right for comparison.

**Atomic Force Microscopy:** The dispersity of MinEx and lab-scale extrudates was analysed with tapping mode AFM. The molecular roughness of exposed fracture surfaces was assessed complementary with phase maps as established previously [7,8]. Following established structural-morphologic criteria, only ASDs which have a root mean square roughness (Rq) smaller or similar to those measured on extrudates of pure HPMCAS were here considered disperse on a molecular level. This is the case for all extrudates, except all those containing SA (Figure 5). Extrudates containing SA are rough, also, the phase maps reveal up to square micron sized but ultrathin nanoplatelets. Taken together, the dispersity of lab-scale extrudates quantitatively match with those prototyped with MinEx on a molecular level. SA is found to be intrinsically immiscible at all given conditions, both preparation techniques resulted in a continuously de-mixed system.

### 3.5. Solid State Properties of MinEx Extrudates and Powders Derived from Lab-Scale Extrudates

**Differential Scanning Calorimetry (DSC):** MinEx extrudates were investigated using DSC and compared with powders obtained after milling of lab-scale extrudates (Table 4). Both APIs were initially present in an amorphous form because API melting peaks, which would suggest crystalline particles, were lacking. A single glass transition temperature is observed on all samples except for both MinEx extrudates, as well as on powder derived from CETP lab-scale extrudates containing SA. In these cases an endothermic transition peak as it is expected for SA was observed [35].

**Figure 6:**
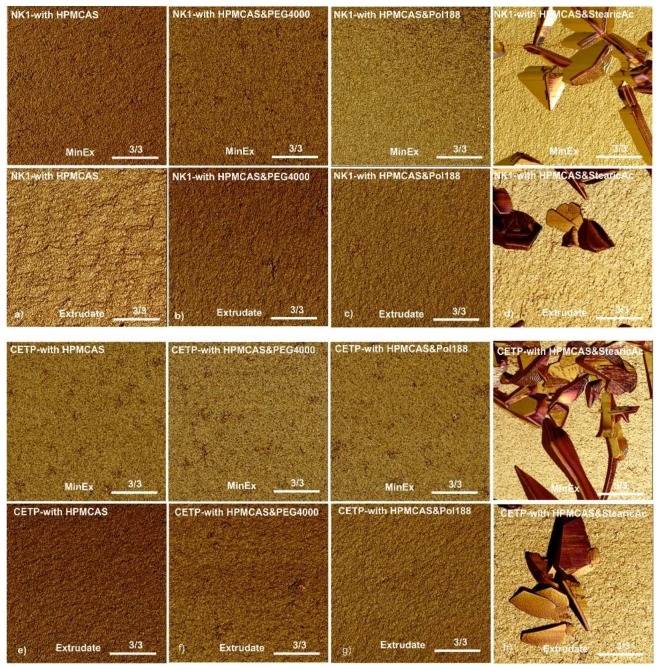
Phase homogeneity maps obtained with tapping mode atomic force microscopy (AFM) on fracture surfaces of NK1 (**a**–**d**) and CETP (**e**–**h**) extrudates. Row 1 shows NK1-MinEx and row 2 shows NK1 lab-scale extrudates. Row 3 shows CETP-MinEx and row 4 shows the CETP lab-scale extrudates. Similarly composed extrudates are grouped in columns. Binary systems with HPMCAS are shown in column 1, with HPMCAS + PEG4000 in column 2, with HPMCAS + Pol188 in column 3 and with HPMCAS + SA in column 4. The scale bar in all images is similar and corresponds to a lateral distance of 2 μm, the numeric ratio shown above indicates the overall reproducibility of the observation. A ratio of 3/3 indicates that the other 2 investigated regions which are not explicitly shown here showed comparable features.

All other samples (no plasticizer/PEG4000/Pol188) show a single glass transition temperature. AFM micrographs of extrudates whether they were prepared with MinEx or lab-scale, so independent of processing, revealed phase heterogeneity in presence of SA (Figure 6), fracture surfaces are in these cases spiked with ultrathin nanoplatelets. However, after milling of lab-scale extrudates, the NK1:HPMCAS:SA systems appeared to be a single phase system in DSC and the system with CETP not.

**Figure 7:**
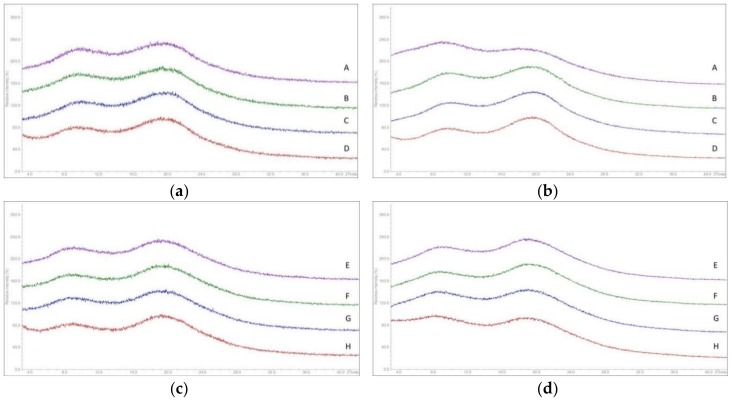
Overlay of X-Ray powder diffraction (XRPD) pattern for extrudates containing NK1 derived from MinEx extrusion (**a**), lab-scale extrusion (**b**) and containing CETP derived from MinEx extrusion (**c**), lab-scale extrusion (**d**). Pink curves are extrudates without plasticizer, green with PEG4000, blue with Pol188, and red curves with SA.

**X-ray Powder Diffraction (XRPD):** XRPD shows that both APIs were present in amorphous form as indicated by the typical halo in the X-ray pattern, see Figure 7. XRPD was not able to detect the presence of ultrathin nanoplatelets of SA, neither in the MinEx extrudate nor in the powders which were derived from lab-scale extrusion trials. 

**Figure 8:**
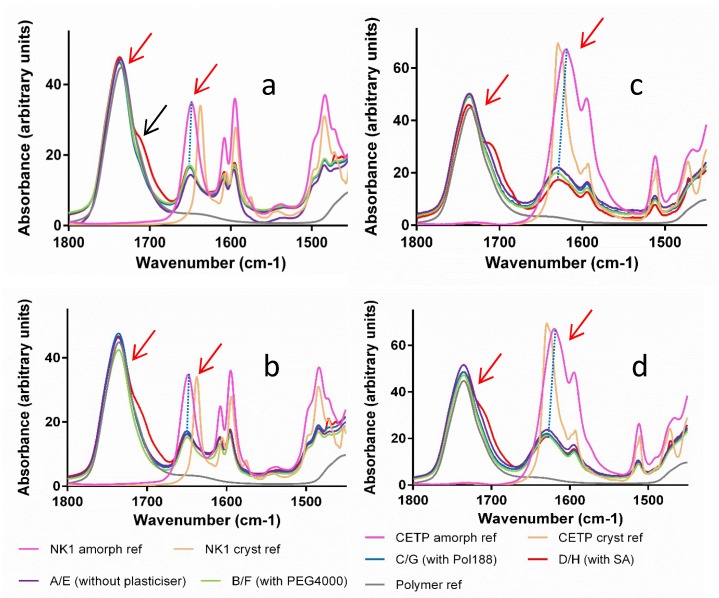
Infrared spectra comparison of amorphous and crystalline reference active pharmaceutical ingredients (API) with extrudates prepared with MinEx and lab-scale extruder, in presence and absence of plasticizers. The left column shows series of NK1 mixtures derived from MinEx extrudates (**a**), those from lab-scale extrusion are shown in (**b**). The CETP series is shown on the right hand side, derived from MinEx and (**c**) from lab-scale extruder in (**d**). The red arrows show the position of carbonyl stretching vibrations for the polymer (**left**) and NK1/CETP (**right**). The black arrow shows the carbonyl stretching vibrational band from SA.

**Fourier Transform Infrared Spectroscopy (FTIR):** The hydrogen bonding network of the APIs, HPMCAS and plasticizers was studied by FTIR spectroscopy. The measurements were conducted using an attenuated total reflection (ATR) accessory. Absorption bands related to the carbonyl hydrogen bond acceptor groups typical for the polymer, ν ~ 1740 cm^−1^ and for the API, ν ~ 1640 cm^−1^, were assessed for comparison between the different mixtures (Figure 8—red arrows). From the spectra, it is clear that the differences between materials derived from MinEx and lab-scale extrudates are overall small and all extrudates contain the API in a non-crystalline form. The presence of Pol188 and PEG4000 is not visible in the depicted interaction region. SA is visible as a pronounced shoulder on the right-hand side of the 1700 cm^−1^ polymer carbonyl peak (see black arrow), albeit shifted to higher wavenumbers compared to the SA reference spectrum. 

When assessing the NK1 and CETP carbonyl group, compared to amorphous API reference spectra, a small shift occurs towards less hydrogen bonding (see blue dashed line) due to spatial competition of intermolecular API-API interactions with the much more abundant polymer.

### 3.6. Dissolution Properties of MinEx Extrudates and Powders Derived from Lab-Scale Extrusion

**Figure 9:**
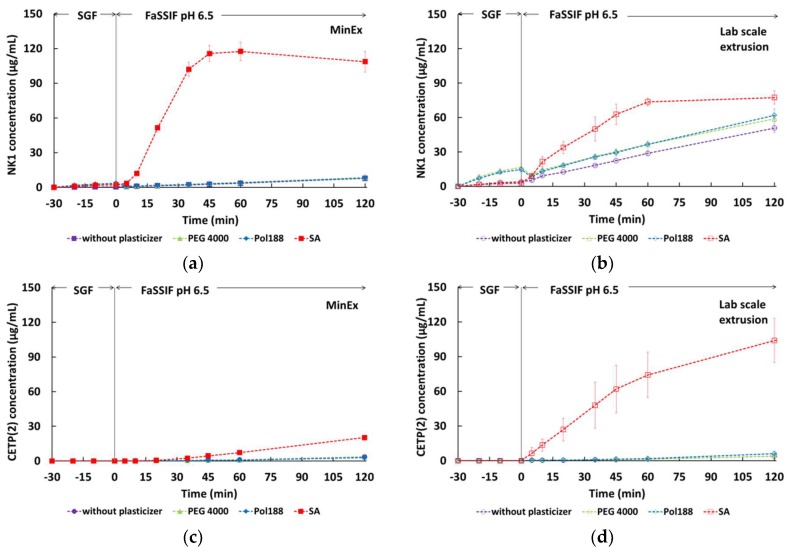
Two-stage dissolution profiles during initial analysis for extrudates containing NK1 derived from MinEx extrusion (**a**), lab-scale extrusion (**b**) or containing CETP derived from MinEx extrusion (**c**), lab-scale extrusion (**d**).

MinEx extrudates containing a total of 6 mg API were used for two-stage dissolution studies (Figure 9). The two-stage dissolution test mimicked the transit from the stomach (pH 2) into the intestinal (pH 6.5) fluids through a medium change after 30 min. In both cases significant drug release was only observed in the intestinal stage of the dissolution test, which was expected due the pH-dependent solubility of HPMCAS (pH ≥ 5.5). A linear release profile was observed for NK1 formulations without plasticizer as well as for formulations containing either PEG4000 or Pol188, which resulted in a total release of 8 to 8.6 μg/mL after 2 h in fasted state simulated intestinal fluid (FaSSIF). In contrast, the NK1 formulation containing SA showed a fast release during the first hour, which levelled off at around 120 μg/mL. A similar formulation ranking was observed for CETP samples showing almost linear release in all cases and faster release in presence of SA. The concentration of CETP dissolved after 2 h of dissolution testing was 2.8 to 3.6 μg/mL for the extrudate without plasticizer, with PEG4000 and Pol188 and 20 μg/mL for the extrudate with SA.

Similar to the characterization of MinEx extrudates, the dissolution behaviour of the milled lab-scale extrudates was investigated (Figure 9). In case of NK1 the final concentration of drug dissolved from the crystalline reference was ~3 μg/mL after 2 h in FaSSIF compared to more than 60 μg/mL for the extrudates containing amorphous drug. The milled NK1 extrudates exhibited the following ranking with respect to dissolution behaviour: extrudates without plasticizer < extrudates with PEG4000 = extrudates with Pol188 < extrudates with SA. The extrudates containing SA showed most extensive release reaching a final concentration of 77 μg/mL. This effect was even more pronounced with samples containing CETP. After 240 min in FaSSIF more than 130 μg/mL CETP were released from the extrudate containing SA, whereas all other extrudates resulted in a final concentration of 13 to 16 μg/mL. Visual observations made during dissolution testing indicated that in contrast to the other extrudates, which formed a coarse suspension, the extrudates containing SA formed a fine suspension with gradually disintegrating particles. 

Summarizing, the MinEx and powders of milled lab-scale extrudates show comparable release profiles as long as SA is absent. Pol188 and PEG4000 do not really modulate the dissolution behaviour, because the corresponding profiles merge with those of the binary mixtures. Quantitative differences might be caused by the fact that MinEx spaghettis were compared with lab-scale powders. However, the presence of SA non-linearly changes the dissolution behaviour in an unforeseen manner.

### 3.7. Phase Separation Processes on MinEx and Lab-Scale Extrudates (AFM)

**Figure 10:**
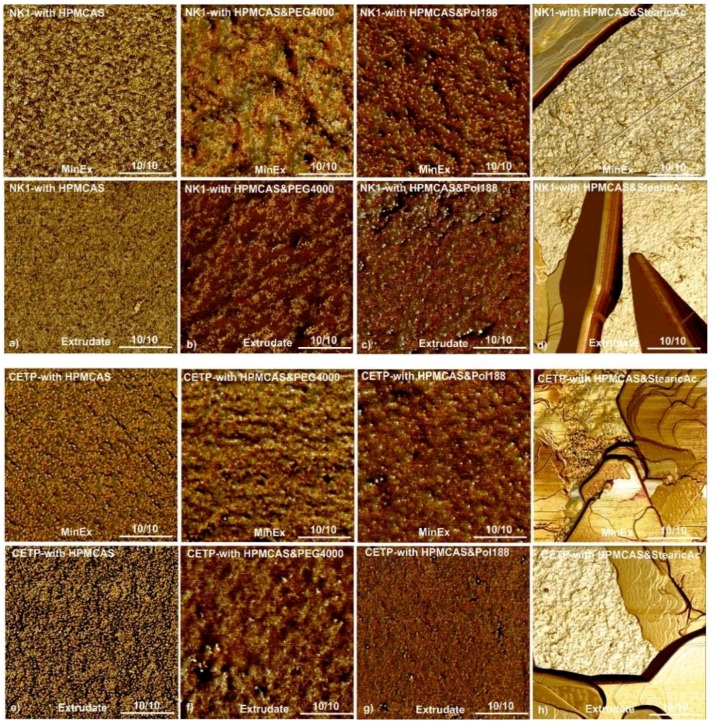
Phase maps recorded with tapping mode AFM on fracture surfaces of extrudates which have been exposed to temperature humidity stress storage for 2 h, *T* = 40 °C and RH = 75%, NK1 series (**a**–**d**) and CETP2 series (**e**–**h**). Row 1 shows NK1-MinEx and row 2 shows NK1-lab-scale extrudates. Row 3 shows CETP-MinEx and row 4 shows the CETP-lab-scale extrudates. Similarly composed extrudates are grouped in columns. Binary systems with HPMCAS are shown in column 1, with HPMCAS + PEG4000 in column 2, with HPMCAS + Pol188 in column 3 and with HPMCAS + SA in column 4. The scale bars in all imagesare identical and correspond to a lateral distance of 1 μm, the numeric ratio shown above indicates the overall reproducibility of the observation. A ratio of 10/10 suggests that the other 9 investigated regions which are not shown here showed comparable features.

The phase maps recorded with tapping mode AFM after humidity-temperature stress storage for 2 h at *T* = 40 °C and relative humidity (RH) = 75%RH were inspected for patterns indicative for de-mixing or phase separation processes. Ten regions on each of the 16 extrudates were inspected and the results are presented in Figure 10. Independent of whether originating from MinEx or the lab-scale processes, whether they contain NK1 or CETP, all systems which contained PEG4000 and Pol188 were poorly stable, because spinodal nucleation patterns were detected. Extrudates which contain SA or which lack plasticizers were stable. 

### 3.8. Macroscopic Physical Stability of Lab-Scale Extrudates with XRPD, DSC and FTIR.

The physical long-term stability of powders derived from milled lab-scale extrudates was explored using DSC and XRPD, selected samples were investigated using FTIR spectroscopy (Table 5).

**Figure 11:**
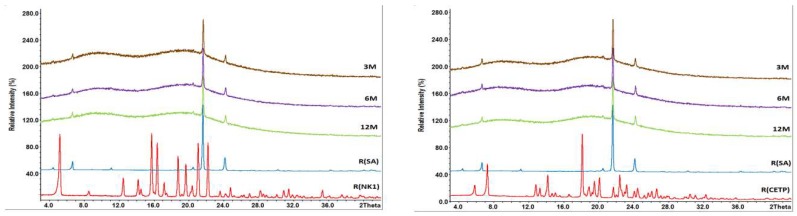
Overlay of XRPD pattern of milled extrudates derived from lab-scale extrusion containing SA. NK1 (**left**) and CETP (**right**) samples stored for 3 months (3M), 6 months (6M) and 12 months (12M) at 40 °C/75%RH. The reference spectra of crystalline NK1 (R(NK1)), of crystalline CETP (R(CETP)) and stearic acid (R(SA)) are given as well.

Based on XRPD and DSC, no re-crystallization of the API was observed after 12 months open storage even at 40 °C/75%RH for the extrudates containing HPMCAS without plasticizers or with SA as plasticizer. The samples containing SA show distinct peaks but they do not match XRPD patterns of the APIs. They are attributed to crystalline SA and occurred already at the first stability time point after 3 months as shown in Figure 11.

**Figure 12:**
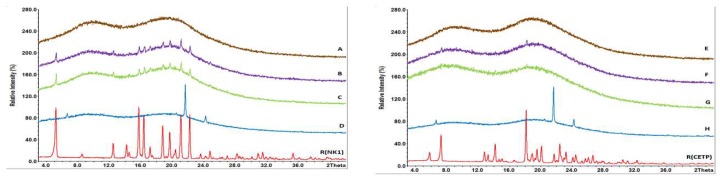
Overlay of XRPD pattern of milled extrudates derived from lab-scale extrusion containing NK1 ((**left**) (**A**–**D**) and crystalline reference R(NK1)) or CETP ((**right**) (**E**–**H**) and crystalline reference R(CETP)), which were stored for 12 months at 40 °C/75%RH. Extrudates without plasticizers are shown in brown (**A**,**E**), with PEG4000 in pink (**B**,**F**), with Pol188 in green (**C**,**G**), and with SA in blue (**D**,**H**). The crystalline reference spectra are shown at the bottom with red curves.

However, crystalline traces of NK1 were found already after 3 months in the samples containing either PEG4000 or Pol188 stored at 40 °C/25%RH or 40 °C/75%RH. Upon further storage, the amount of crystalline material increased. The X-ray pattern of the samples stored for 12 months at 40 °C/75%RH are shown in Figure 12. A comparison with the reference spectra of crystalline NK1 showed that the peaks occurring in formulation B and C correspond to the crystalline drug substance. The milled extrudates containing NK1 were quite stable at 25 °C/60%RH with the exception of the sample containing PEG4000. Here first traces of the crystalline API were detected after 12 months storage. 

Similar results were also obtained for the extrudates containing CETP. No re-crystallization of the API was observed after 12 months open storage at 40 °C/75%RH for binary extrudates or extrudates with SA (see Figure 12). Crystallization was observed on the extrudates containing PEG4000 or Pol188 after already 3 months. Crystallization of the API was also noticed at lower stress conditions, such as 40 °C/25%RH after 3 months or 25 °C/60%RH after 12 months. Further analysis of the data showed that PEG4000 and Pol188 did not crystallize during storage, whereas SA is present in the crystalline state as seen from the X-ray overlays in Figure 11. Based on the results of XRPD and DSC the amount of crystalline Form C (CSD Ref code: STARAC01 [36]) of SA is increasing over time.

Taken together the long-term stability of all systems match the ranking and observations made on fracture surfaces of MinEx and lab-scale extrudates after already 2 h with AFM (Figure 10). The presence of Pol188 and PEG4000 destabilizes both systems and crystals become detectable with X-ray on shorter time scales. It can further be stated that the milling process might have reduced the crystallinity of SA, upon storage novel crystals in any case evolve. The absence of crystalline SA after milling in lab-scale extrudates was also indicated with XRPD (Figure 7).

**Figure 13:**
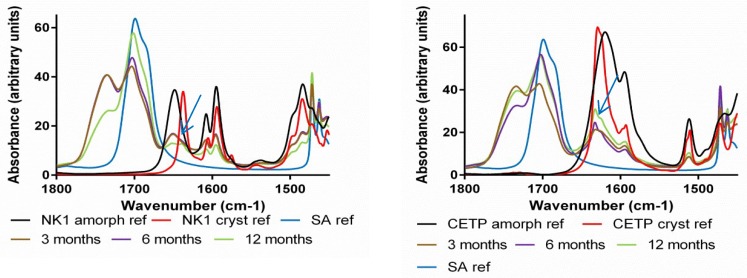
Attenuated total reflection (ATR) FTIR spectra of milled extrudates derived from lab-scale extrusion containing SA. NK1 (**left**) and CETP (**right**) samples stored for 3, 6 and 12 months at 40 °C/75%RH. The reference spectra of crystalline and amorphous NK1, of crystalline and amorphous CETP and SA are given as well. The blue arrows show a crystalline motif observed at the position of the NK1 or CETP carbonyl stretching vibration.

**Figure 14:**
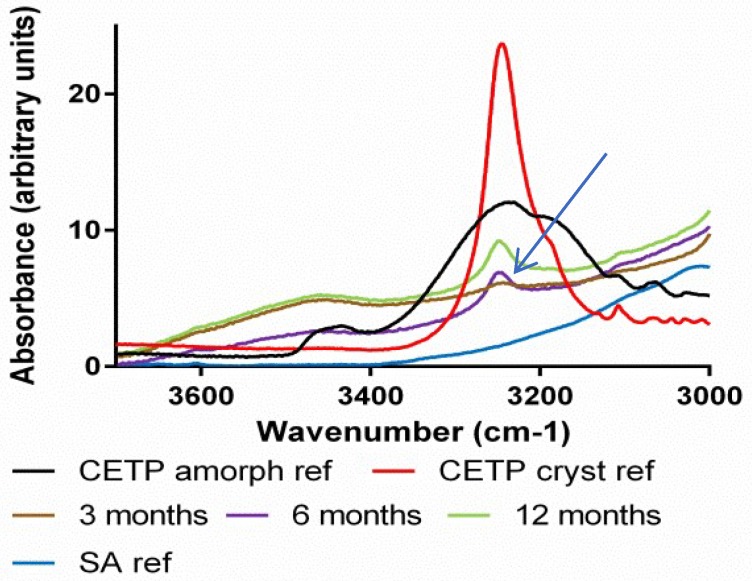
ATR FTIR spectra of milled extrudates derived from lab-scale extrusion containing SA featuring the CETP NH stretch region. CETP samples initial (I) and stored for 3 months (3M), 6 months (6M) and 12 months (12M) at 40 °C/75%RH. The reference spectra of crystalline CETP and amorphous CETP are given as well. The blue arrow shows a crystalline motif observed at the position of the CETP amine N–H stretching vibration.

**Figure 15:**
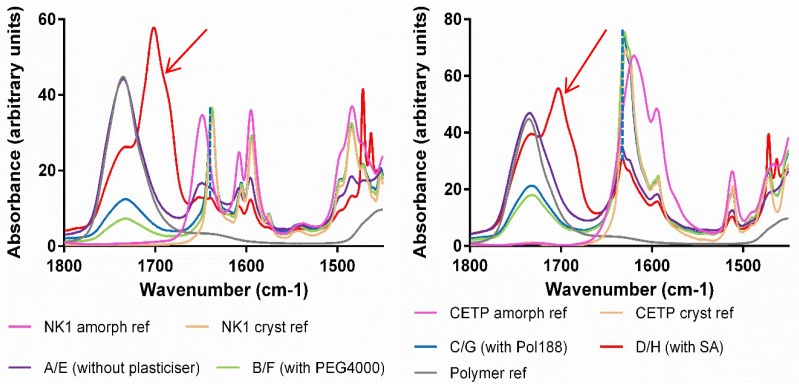
ATR FTIR spectra of milled extrudates derived from lab-scale extrusion containing NK1 (**left**) and CETP (**right**), which were stored for 12 months at 40 °C/75%RH. The red arrows show the presence of SA.The blue dashed line shows that even after 12 months at 40 °C/75%RH, the API carbonyl stretching frequencies don’t perfectly overlap with those in the reference spectrum of crystalline NK1 or CETP.

With FTIR spectroscopy changes within the supramolecular order and physical interaction of APIs, HPMCAS and plasticizers were followed during stress storage at various humidity temperature conditions and over a time span of up to 12 months (Figure 13, Figure 14 and Figure 15). The spectroscopic method assesses qualitative changes of the intermolecular hydrogen bonding network, because band shifts, as they accompany rearrangements of functional groups and conformational changes influence the absorption wavelengths and vibrational band intensities of the material [2]. The IR spectra of NK1 and CETP were initially measured for reference and then provided a rationale to compare and assess the presence/absence of amorphous-/crystalline-like short range order during the stress tests. 

To verify the possible onset of API crystallization we focused on band shifts related to the carbonyl groups (red arrow around 1640 cm^−1^ in Figure 8), because these peaks are specific for crystalline and amorphous interaction motifs and for both APIs.

Taken together the IR results confirm all trends observed with XRPD and DSC, materials which contain PEG4000 and Pol188 are less stable. For the extrudates without plasticizer and SA, crystalline packing motifs occurred after 12 months at 40 °C/75%RH but the API carbonyl peak does not completely match with the reference spectrum of crystalline material, some amorphous part remains (see blue dashed line in Figure 15).

In some cases, the IR spectra revealed crystalline motifs at an earlier time point than XRPD and/or DSC. Crystalline-like IR interactions were observed even in binary CETP extrudates, or also in ternary compositions with SA and already after 6 months storage at 40 °C/75%RH (Table 5). Furthermore, the CETP extrudate with SA was found to have changed after 3 months storage at *T* = 40 °C, RH = 75%RH. This is documented by changes in the absorption bands in the carbonyl and amine stretching regions, as depicted in Figure 13 and Figure 14 respectively, with blue arrows. However, all these mixtures are found to be amorphous after 12 months by XRPD and DSC, which indicates that the mobility of the API in the HPMCAS matrix is not sufficient to enable growth of detectable crystals, as long as Pol188 and PEG4000 are absent. 

Taken together the IR study reveals that the physical strength of the API-polymer carrier matrix changes in the presence of humidity and temperature stress storage. Rearrangements towards crystalline packing motifs occur in nearly all mixtures, enhanced under long-term high humidity stress conditions. However, the mobility of the APIs in the HPMCAS carrier in absence of hydrophilic compounds, PEG4000 and Pol188, stays low. Well-separated and bulky crystalline API domains which would be detectable with DSC or XRPD are then absent, even after 12 months.

## 4. Discussion

Plasticizer selection is a multivariate challenge. Compounds which beneficially lower the melt viscosity and reduce the chemo-mechanical stress of the extrusion processes can impair the physical long-term stability of the product, modulate dissolution behaviour or worsen the downstream processability of a basically promising dispersion system [37,38,39]. Therefore, the selection of suitable plasticizers requires the same attention as more established polymer carrier systems [4,39]. In this context our prototyping device, MinEx applied in combination with torque rheometry, is a promising and elegant approach with predictive value. 

The physico-chemical properties of materials prototyped with MinEx resemble extrudates manufactured with the lab-scale process; if differences can be noticed at all they are small. 

All extrudates with SA, being prepared with the lab-scale process or prototyped with MinEx, are phase separated on nanometre scales. The miscibility of SA in binary API:HPMCAS matrices is poor. SA is found condensed in ultrathin but square micrometre-sized platelets as visualized with AFM (Figure 6). Thermal material properties measured with DSC also indicate SA to be present in separated phases, on both MinEx extrudates and also on the powder derived from the lab-scale NK1 process. The phases detected are poorly ordered because bulky and coherently ordered crystals were not observed with XRPD. The SA platelets are only few nanometres thin. Furthermore, it cannot be excluded that the applied downstream process has, maybe partly, transformed the dispersity and/or crystallinity of SA. SA is a soft and waxy material and has a low melting point. As other fatty acids it tends to assemble in lamellar bilayers [35].

SA modulates the viscosity–temperature profile of NK1 and CETP2 mixtures in a comparable manner. Once the melting temperature of SA is exceeded, the torque decreases (Figure 4). Both API compositions are then likewise easy to shear, also, less energy is required at steady state if compared with binary mixtures. Overall, the temperature-viscosity profiles qualitatively impose or still remind on those of binary compositions, however, they appear shifted to lower temperatures and energies. The presence of thin and liquid layers might introduce lamellar-waxy slip planes which lower the friction between micrometre domains of the more viscous and binary API:polymer phases. 

SA does not modulate the physical stability of both ASDs upon long-term humidity storage (Figure 11 and Figure 12). The materials with SA perform like two independent and separated phases. Interaction analysis based upon FTIR spectroscopy, however, indicates that both APIs only weakly interact with the polymer carrier matrix, HPMCAS (Figure 8). Storage at high humidity conditions triggers rearrangement of amorphous towards crystalline packing motifs, similarly as in binary mixtures (Figure 13, Figure 14 and Figure 15). However, the API mobility is small, the APIs are found to be trapped in a structurally persistent carrier. Therefore, phase separation processes on surfaces of stressed binary mixtures and mixtures with SA are not observed on molecular length and time scales (Figure 10). Moreover, high humidity stress storage over the time range of a year does not enable the formation of a sufficiently sized bulk phase, which would be detectable with DSC and/or XRPD (Table 5).

The systems containing SA, independent of whether they are produced with MinEx or powders derived with the lab-scale process, show extensive release in FaSSIF (Figure 9). Visual observations made during our testing indicate that systems with SA form a likewise fine suspension with gradually disintegrating particles. SA is found dispersed in ultrathin and lamellar structured layers in the solid state (Figure 6). The dynamic and microscopic scenarios of dissolution processes in gastrointestinal media depend on many factors and are known to be complex [40]. However, being a poorly soluble fatty acid, SA is likely to transition into mixed micellar aggregates in presence of bile salts or FaSSIF [41,42]. Emulsification would explain the rapid disintegration of the ASDs and also enlarge the lipophilic volume of the dissolution medium, the capacity to trap and stabilize NK1 or CETP is in any case observed to be larger. Comparable concepts have been explored in the past on solid lipid formulations [43,44] and dissolution studies which have been conducted on extruded solid lipid formulation systems in bio relevant media show a trend to be complex and to depend on many factors [45].

The extrudates with Pol188 and PEG4000 are different. Independent of whether they have been prepared with MinEx or with the lab-scale extruder, all amorphous mixtures are molecular disperse (Figure 5 and Figure 6). Pol188 and PEG4000 are better miscible within both ASDs and therefore homogenously distributed, a single phase ASD is observed (Table 4). 

Pol188 and PEG4000 beneficially modulate the viscosity-temperature profile. Interestingly, all profiles appear comparable from a quantitative point of view (Figure 4). Being better soluble in both binary mixtures, they are expected to have a different mode of plasticizing action than SA, they are primary plasticizers [14]. Once Pol188 or PEG4000 are molten other components start to dissolve in a steadily present fluidic-viscous phase and this phase is expected to systematically grow with temperature and mixing shear. 

In contrast to SA, PEG4000 and Pol188 are better soluble in water and therefore expected to increase the diffusion and permeability of moisture in amorphous matrices. This effect has already been studied on thin films of HPMCAS and PEG400 [46]. The presence of water lowers the Tg of the amorphous system and reduces the kinetic stability of the matrix. It is clear that moisture drives phase separation processes of hydrophobic compounds. ASDs containing PEG4000 and Pol188 are mobile on molecular scales (Figure 10) and they have insufficient storage stability in moisture at the long-term, which is shown by the detection of crystals of NK1 and CETP with XRPD (Figure 11 and Figure 12).

## 5. Conclusions

MinEx in combination with torque rheometry enables to properly assess the impact of plasticizers on processability and performance within only hours. The complementarity of analytical methods which probe different physical properties on various length scales enables to enlighten structure and property relations of complex materials on a molecular-mechanistic level, distinctions between different modes of plasticizing actions can be made. On the first view SA appears to be the most promising plasticizer of our study. In contrast to PEG4000 and Pol188 it does not reduce the storage stability of both products. Also, SA boosts the dissolution and/or disintegration of both ASDs in an unforeseen manner. However, the dissolution tests point to a likewise high degree of variability and most likely depend on downstream processing aspects, more experiments will be required to confirm and understand the identified trends in detail.

## Figures and Tables

**Table 1:** Composition of physical mixtures (used in the torque rheometer) and extrudates (w %).

Formulation	NK1	CETP	HPMCAS *	PEG4000	Pol188 **	SA ***
A: NK1 + HPMCAS	30.0		70.0			
B: NK1 + HPMCAS + PEG4000	27.8		65.0	7.2		
C: NK1 + HPMCAS + Pol188	27.8		65.0		7.2	
D: NK1 + HPMCAS + SA	27.8		65.0			7.2
E: CETP + HPMCAS		30.0	70.0			
F: CETP + HPMCAS + PEG4000		27.8	65.0	7.2		
G: CETP + HPMCAS + Pol188		27.8	65.0		7.2	
H: CETP + HPMCAS + SA		27.8	65.0			7.2

* Mixture of LF and LG (30:70) was used; ** Poloxamer 188; *** stearic acid.

**Table 2:** High performance liquid chromatography (HPLC) methods used for drug quantification. NK1: neurokinin-1 receptor antagonist; CETP: cholesterylester transfer protein.

HPLC Parameter	NK1	CETP
Sample temperature	25 °C	25 °C
Mobile phase	60% Acetonitrile/40% Water/0.1% TFA	80% Acetonitrile/20% Water/0.1% TFA
Flow rate	1.0 mL/min	1.0 mL/min
Elution time	5 min	4 min
Injection volume	10 mL	10 mL

**Table 3:** Comparison of the *SE*_SS_ from torque rheometer and specific mechanical energy (SME), pressure and temperature at die from lab-scale extrusion ^a^.

Formulation	Torque Rheometer	Lab-Scale Extrusion ^b^		
	*SE*_SS_ (kJ/kg)	SME (kJ/kg)	Pdie ^c^ (bar)	Tdie ^d^ (°C)
A: NK1 + HPMCAS	683	6654	11.9 ± 2.8	157–161
B: NK1 + HPMCAS + PEG4000	305	4616	6.2 ± 1.6	157
C: NK1 + HPMCAS + Pol188	324	3714	5.5 ± 1.6	157
D: NK1 + HPMCAS + SA	263	3462	4.7 ± 1.3	157
E: CETP + HPMCAS	525	5042	10.4 ± 1.9	159–163
F: CETP + HPMCAS + PEG4000	250	3082	6.1 ± 1.1	157–159
G: CETP + HPMCAS + Pol188	260	3053	6.0 ± 1.1	157–160
H: CETP + HPMCAS + SA	222	2745	5.4 ± 0.9	157–160

^a^*SE*_SS_ specific energy at steady state, SME specific mechanical energy, ^b^ using the Brabender Lab-scale TSE 12/36, ^c^ pressure at die, ^d^ temperature at die.

**Table 4:** Comparison of Differential Scanning Calorimetry (DSC) data from MinEx and lab-scale extrusion.

Formulation	MinEx Extrusion	Lab-Scale Extrusion ^a^
A: NK1 + HPMCAS	Single *T*g (67 °C)	Single *T*g (59 °C)
B: NK1 + HPMCAS + PEG4000	Single *T*g (60 °C)	Single *T*g (49 °C)
C: NK1 + HPMCAS + Pol188	Single *T*g (64 °C)	Single *T*g (50 °C)
D: NK1 + HPMCAS + SA	*T*g (54 °C), *T*m (68 °C)	Single *T*g (46 °C)
E: CETP + HPMCAS	Single *T*g (54 °C)	Single *T*g (55 °C)
F: CETP + HPMCAS + PEG4000	Single *T*g (49 °C)	Single *T*g (53 °C)
G: CETP + HPMCAS + Pol188	Single *T*g (56 °C)	Single *T*g (47 °C)
H: CETP + HPMCAS + SA	*T*g (53 °C), *T*m (65 °C)	Tg (52 °C), *T*m (64 °C)

**^a^** Using the Brabender Lab-scale TSE 12/36.

**Table 5:** Physical stability of extrudates containing NK1 and CETP (A—Amorphous, C—crystallinity, first letter stands for DSC and second letter for XRPD results) c—onset of crystallization and amorphous by Fourier transform infrared (FTIR) (data not collected for all time points).

Storage Condition	25 °C/60%RH			40 °C/25%RH			40 °C/75%RH		
Storage Time (Months)	3	6	12	3	6	12	3	6	12
A: NK1 + HPMCAS	AA	AA	AA	AA	AA	AA	AAa	AAa	AAc
B: NK1 + HPMCAS + PEG4000	AA	AAc	CC	CAc	CCc	CC	CCc	CCc	CCc
C: NK1 + HPMCAS + Pol188	AA	AAc	AA	CAc	CCc	CC	CCc	CCc	CCc
D: NK1 + HPMCAS + SA	AA	AA	AA	AA	AA	AA	AAa	AAc	AAc
E: CETP + HPMCAS	AA	AA	AA	AA	AA	AA	AAa	AAc	AAc
F: CETP + HPMCAS + PEG4000	AA	AAa	CA	CAc	CCc	CC	CCc	CCc	CCc
G: CETP + HPMCAS + Pol188	AA	AAa	CA	CAc	CCc	CC	CCc	CCc	CCc
H: CETP + HPMCAS + SA	AA	AA	AA	AA	AA	AA	AAc	AAc	AAc
